# Gout and Migraines: Exploring the Complex Link in a 16-Year Longitudinal Study of the Korean Population

**DOI:** 10.3390/jcm13010138

**Published:** 2023-12-26

**Authors:** Ho Suk Kang, Ji Hee Kim, Joo-Hee Kim, Woo Jin Bang, Hyo Geun Choi, Nan Young Kim, Ha Young Park, Kyung Chan Choi, Younghee Choi, Mi Jung Kwon

**Affiliations:** 1 Division of Gastroenterology, Department of Internal Medicine, Hallym University Sacred Heart Hospital, Hallym University College of Medicine, Anyang 14068, Republic of Korea; hskang76@hallym.or.kr; 2 Department of Neurosurgery, Hallym University Sacred Heart Hospital, Hallym University College of Medicine, Anyang 14068, Republic of Korea; kimjihee.ns@gmail.com; 3 Division of Pulmonary, Allergy, and Critical Care Medicine, Department of Medicine, Hallym University Sacred Heart Hospital, Hallym University College of Medicine, Anyang 14068, Republic of Korea; luxjhee@gmail.com; 4 Department of Urology, Hallym University Sacred Heart Hospital, Hallym University College of Medicine, Anyang 14068, Republic of Korea; yybbang@hallym.or.kr; 5 Suseo Seoul E.N.T. Clinic and MD Analytics, 10, Bamgogae-ro 1-gil, Gangnam-gu, Seoul 06349, Republic of Korea; mdanalytics@naver.com; 6 Hallym Institute of Translational Genomics and Bioinformatics, Hallym University Medical Center, Anyang 14068, Republic of Korea; honeyny78@gmail.com; 7 Department of Pathology, Busan Paik Hospital, Inje University College of Medicine, Busan 47392, Republic of Korea; hy08.park@gmail.com; 8 Department of Pathology, Chuncheon Sacred Heart Hospital, Hallym University College of Medicine, Chuncheon 24253, Republic of Korea; kcchoi@hallym.or.kr; 9 Department of Pathology, Hallym University Dongtan Sacred Heart Hospital, Hallym University College of Medicine, Hwaseong 18450, Republic of Korea; yhchoi@hallym.or.kr; 10 Research Insititute for Complementary & Alternative Medicine, Hallym University, Chuncheon 24252, Republic of Korea; 11 Department of Pathology, Hallym University Sacred Heart Hospital, Hallym University College of Medicine, Anyang 14068, Republic of Korea

**Keywords:** gout, migraine risk, chronic disorder, longitudinal follow-up study, national health screening cohort database

## Abstract

Despite the growing prevalence of gout and its associated health concerns as a chronic disorder, population-based studies on its link to migraines are scarce. We conducted a 16-year longitudinal study in a Korean population to investigate the relationship between gout and migraines, including different subtypes. We enrolled 23,137 patients with gout and matched them with 92,548 controls based on age, sex, income, and residence. Using Cox proportional hazards models, we calculated hazard ratios to assess the likelihood of migraines while considering relevant factors. During the follow-up, 1000 gout patients and 3214 controls experienced migraines. After adjusting for various factors, including demographics, health-related variables, and weight categories, the gout group had a 1.26-fold higher likelihood of developing migraines compared to the group without gout. This association was particularly strong for migraines without aura, while it was not significant for migraines with aura. In summary, our study reveals a significant link between gout and migraines in the Korean population, emphasizing the complex relationship among chronic disorders, with a specific focus on migraine subtypes.

## 1. Introduction

Gout, the predominant type of inflammatory arthritis, is characterized by the progressive accumulation of monosodium urate crystals in the joints and throughout the body, significantly impacting patients’ quality of life and productivity [1]. Globally, gout has become a growing health concern, with a prevalence ranging from 2.49 to 6.7% [1,2,3,4]. The rise in gout prevalence is a global phenomenon that can be attributed to several factors, including the aging population, increasing rates of obesity, and the prevalence of metabolic diseases [2]. Similarly, the specific context of South Korea has contributed to this trend. Over recent decades, shifting demographics and evolving lifestyle patterns in South Korea have led to a significant surge in the incidence of gout [5]. Notably, the prevalence of gout in Korea has experienced a substantial increase, rising from 0.39% in 2002 to 2.01% in 2015 [5]. This increase has translated into a 10.8% annual average rise in healthcare expenses associated with gout [6]. Gout, characterized by elevated uric acid levels, often coexists with a range of comorbidities [3], including hypertension, obesity, diabetes mellitus, chronic kidney disease, osteoporosis, cardiovascular diseases, and neurodegenerative disorders [7,8,9,10,11,12]. Consequently, gout and the comorbid conditions associated with it are increasingly recognized as significant public health concerns in South Korea.

Migraines are the second most prevalent disabling neurological disorder globally, affecting approximately one in ten individuals, with a higher prevalence among those aged 25–64, particularly among women and urban dwellers [13,14]. This condition is characterized by recurrent, often excruciating, unilateral throbbing headaches and is sometimes associated with symptoms such as nausea, light sensitivity, and sound sensitivity [15]. Migraines are categorized into two primary subtypes: migraines without aura and migraines with aura [13]; the latter includes visual, sensory, or other central nervous system symptoms that precede the onset of the headache [13]. While the pathophysiology of the migraines results from intricate interactions among neurons, glial cells, the vasculature, and inflammatory signaling [16], previous studies have reported it can develop as a complication of inflammatory arthritis [17,18] or autoimmune disorders like rheumatoid arthritis [19]. Furthermore, recent epidemiological research has revealed gouty arthritis is associated with an increased risk of migraine occurrence [20]. This potential link between arthritis and migraines may be attributed to the activity of systemic inflammation and oxidative stress, which could potentiate the neurogenic inflammation associated with migraines [17,18,20,21]. Furthermore, gout is associated with a range of comorbidities, including cardiovascular diseases, stress, anxiety, depression, and sleep apnea, which are consistent with the comorbidities contributing to migraine development [14,22]; this indicates they may share contributing factors.

In light of the well-understood etiopathogenesis of gout, with hyperuricemia as a primary intermediary step, attention has been drawn to the repeated observations of a potential link between serum uric acid levels and migraine occurrence [23,24,25,26]. For instance, a cross-sectional cohort study revealed an exponential relationship between serum urate levels and migraine occurrence when serum urate exceeded 7.8 mg/dL [24]. A positive relationship exists between variations in serum uric acid levels during migraine attacks and the intensity of pain [23]. These findings suggest elucidation of the possible relationship between gout and migraine. However, the studies referenced are indeed cross-sectional in nature [23,24,25,26], and they do not encompass follow-up data [27]. This underscores the need for further research employing national population cohort data, which includes meticulous demographic matching and long-term follow-up, to replicate and substantiate the potential link observed in these studies.

The primary objective of this study was to evaluate whether patients with gout in the Korean population might exhibit an elevated likelihood of developing migraines while carefully controlling for various potential confounding factors. To investigate the potential link between gout and migraine onset, we balanced the baseline characteristics between the patient and control groups and conducted a 16-year nationwide database surveillance analysis.

## 2. Patients and Methods

This study utilized the Korean National Health Insurance Service-Health Screening Cohort (KNHIS-HSC) database, which is a comprehensive resource for policy and academic research, with anonymized data and diagnostic codes adhering to the International Classification of Diseases, Tenth Revision, Clinical Modification (ICD-10-CM) guidelines. Since the Korean National Health Insurance Service (KNHIS) had expanded compulsory health insurance coverage to encompass approximately 97% of the population starting in 1999, the KNHIS-HSC sample cohort initially consisted of individuals aged 40–79 years (as of 2002) who underwent health screenings in 2002 and 2003 and was followed up until 2019 [28]. This cohort was composed of 514,866 individuals selected through a 10% simple random sampling method from the pool of health screening participants through the years [28]. The study received ethical approval from the institutional ethics committee (approval No. 2019-10-023) and was granted a waiver for written informed consent due to its utilization of secondary data.

From a pool of 514,866 participants aged ≥40 years, who generated a total of 895,300,177 medical records and had at least two clinic visits between 2002 and 2019, we identified individuals with gout using the ICD-10 code M10 (*n* = 27,313). To focus on newly diagnosed gout cases, we excluded those diagnosed in 2002 (*n* = 2470) to ensure a 1-year washout period. Furthermore, individuals lacking documented blood pressure levels (*n* = 1) and those with pre-existing migraine diagnoses before gout (*n* = 1705) were excluded.

Participants that were enrolled in the control group did not correspond to the gout group between 2002 and 2019 (*n* = 487,553). People in the control group were excluded if they had been assigned the ICD-10 code M10 (gout) once (*n* = 758).

To enhance comparability between gout and control groups, we conducted propensity score matching involving pairing individuals with gout with control participants sharing similar propensity scores based on age, sex, income, and residence. To minimize selection bias, participants in the control (without gout) group were randomly selected in sequential order.

For each participant with gout, the index date was set as the day of their initial gout diagnosis (M10) in the medical insurance database. The same index date was applied to matched participants in the control group. Individuals who had died or were diagnosed with migraines before the index date were excluded from the analysis, resulting in the exclusion of 394,247 individuals from the control group during the matching process.

**Figure 1:**
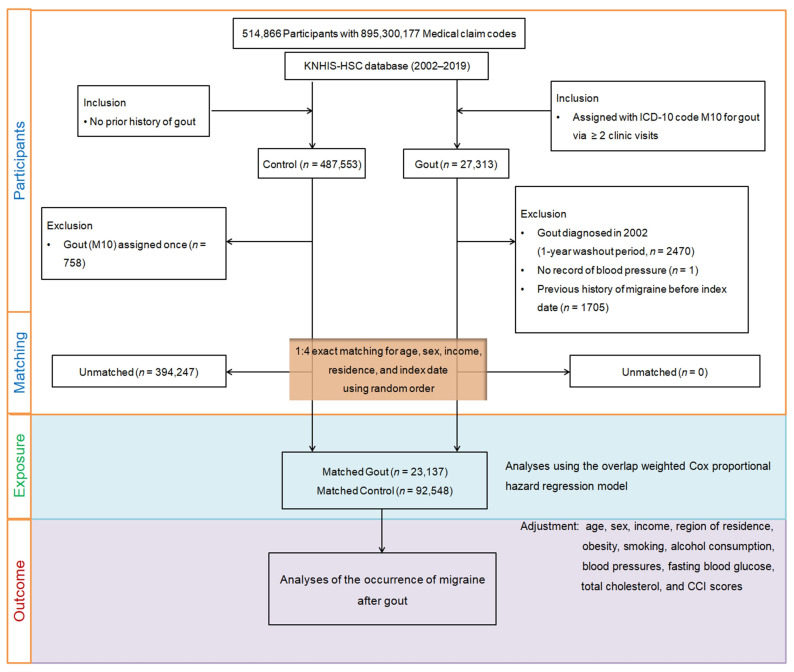
Diagram outlining the participant selection process employed in this study. Out of the initial pool of 514,866 participants in the Korean National Health Insurance Service-Health Screening Cohort (KNHIS-HSC) database, a total of 23,137 patients with gout were carefully matched with 92,548 control participants based on age, sex, income, and region of residence.

Finally, 23,137 participants with gout were matched with four times the number of participants (*n* = 92,548) in the control group. Next, the incidence of newly diagnosed migraine cases was analyzed using ICD-10 codes in gout and control groups from the index date until the end of 2019 (Figure 1).

### 2.1. Definitions of Gout (Independent Variable) and Migraine (Dependent Variable)

Gout (independent variable) was defined as a condition that had been recorded or treated on at least two occasions based on the ICD-10 code (M10), as previously elucidated [5,11].

In the case of migraine (the dependent variable), our definition was based on the ICD-10 code (G43) and whether participants required treatment for symptoms. Migraine patients with a consistent assignment of the ICD-10 code (G43) occurring two or more times were included in the study [29,30]. Among these patients, those diagnosed or treated with the code G431 were categorized as having migraines with aura, while the remaining participants with migraines were grouped as having migraines without aura.

### 2.2. Covariates

We grouped participants into 10 categories (based on age) at 5-year intervals (40–44 to ≥85 years) and classified income into five tiers (class 1–5). Residential areas were divided into two categories: rural, which encompassed various regions based on 16 administrative regions, and urban, which included the seven largest cities in Korea, each with a regional population of >1 million [11]. Participants were categorized into three groups of smoking status, including current smokers, former smokers, and non-smokers. Alcohol intake was divided into <1 time a week or ≥1 time a week. Obesity was determined using body mass index (BMI) in kg/m^2^ and classified into five groups: underweight (<18.5), normal weight (≥18.5 to <23), overweight (≥23 to <25), obese I (≥25 to <30), and obese II (≥30) [31], with additional data recorded for systolic and diastolic blood pressures (mmHg), fasting blood glucose levels (mg/dL), and total cholesterol levels (mg/dL).

To assess the burden of comorbidities, we utilized the Charlson Comorbidity Index (CCI), which quantifies the presence of 17 specific comorbid conditions [32,33]. The CCI score was calculated by summing individual scores for each comorbidity; this yielded a continuous variable ranging from 0 (indicating no comorbidities) to 29 (indicating the presence of multiple comorbidities) [32,33].

### 2.3. Statistical Analyses

We used standardized differences to compare cohort characteristics. Propensity score-based weighting was performed for participants with gout, and the participants in the control group complemented their scores. Propensity scores overlap weighting balanced covariates and optimized sample size, reducing bias risk. An absolute standardized difference ≤0.20 indicated appropriate balance; covariates >0.20 were adjusted using Cox proportional hazards models [34]. Kaplan–Meier analysis and log-rank test compared the cumulative probability of migraine incidence between the gout and control groups. Stratified Cox proportional hazards models with overlap weights were used to assess crude (simple) and adjust hazard ratios (HRs) and 95% confidence intervals (CIs) for gout and migraine, ultimately adjusting for various factors. In these analyses, age, sex, income, and region of residence were stratified.

In our subgroup analyses, we employed stratified and unstratified Cox proportional hazards models to examine various factors; these factors included age, sex, obesity status, fasting blood glucose levels, income level, region of residence, smoking status, alcohol consumption, systolic blood pressure, diastolic blood pressure, total cholesterol levels, and CCI scores.

Furthermore, a *p*-value <0.05 was considered statistically significant, and SAS 9.4 (SAS Institute Inc., Cary, NC, USA) was used in calculating applicable data.

## 3. Results

In this study, 23,137 patients with gout were meticulously matched with 92,548 individuals in the control group. Table 1 presents a comprehensive overview of the baseline characteristics for both groups before and after the application of a weighted propensity score matching to ensure their comparability.

Initially, in the unadjusted analysis, the two groups displayed similar demographic characteristics with a standardized difference of 0, except for the prevalence of overweight or obese individuals. The prevalence of obesity among participants in the gout group was higher than in the control group (73.70% vs. 62.74%). Notably, following the implementation of the overlap weighting modification, these differences were alleviated, leading to standardized differences of <0.2 for all variables; this indicates a more balanced distribution of demographic and health-related attributes between gout and control groups following the adjustment process, which is statistically significant.

### 3.1. Occurrence of Migraines in the Gout and Control Groups

The HRs for the occurrence of overall migraines in patients with gout were summarized, with a follow-up period totaling 170,738 person-years for the gout group and 687,917 person-years for the control group (Table 2).

**Figure 2:**
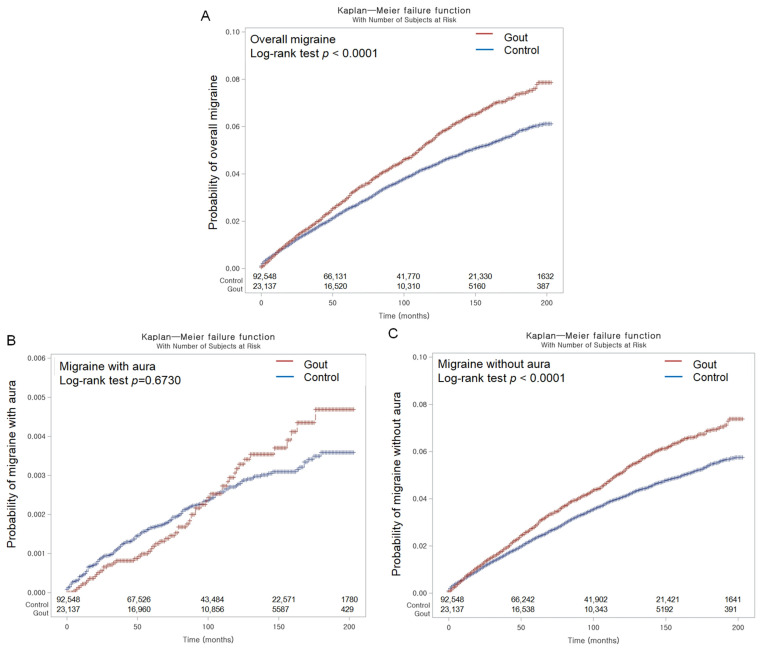
Kaplan–Meier incidence probabilities for overall migraines (**A**) and the subtypes, including migraines with aura (**B**) and migraines without aura (**C**), were assessed in patients with gout and the control populations over 16 years from the index date.

The migraine incidence rates were 5.86 and 4.67 per 1000 person-years in the gout and control groups, respectively. Notably, Kaplan–Meier analysis and log-rank test revealed a higher cumulative probability of incident overall migraines in the gout group than in the control group (*p* < 0.0001; Figure 2A).

Using the Cox proportional hazards model, our analysis indicated that patients with gout had a significantly increased likelihood of developing subsequent migraines compared to the control group; this was detected in the unadjusted analysis (HR 1.25; 95% CI = 1.16–1.34; *p* < 0.001) and after accounting for demographic factors, lifestyle variables, and medical comorbidities (HR 1.26; 95% CI = 1.18–1.33; *p* < 0.001).

Furthermore, in subgroup analyses, the association between gout and an increased likelihood of overall migraines remained significant across various subgroups, which included participants of different sexes, income levels, residential areas, smoking histories, alcohol consumption habits, blood pressure categories, fasting blood glucose levels, total cholesterol levels, and CCI scores. Additionally, all patients with gout under the age of 70 and those with normal weight, overweight, or obesity exhibited an increased likelihood of developing migraines.

### 3.2. Association between Gout and Migraines Based on the Presence or Absence of Aura

We further investigated the relationship between gout and the development of migraines to distinguish between migraines with and without aura.

During the follow-up period, the incidence rates of migraines with aura (0.30 vs. 0.29 per 1000 person-years) were similar between the gout group and the non-gout group (Table 3).

Furthermore, crude and adjusted HRs showed no statistically significant difference, and Kaplan–Meier analysis confirmed similar cumulative probabilities of migraines with aura in both groups (*p* = 0.6730; Figure 2B).

However, for migraines without aura, the incidence rates (5.53 vs. 4.37 per 1000 person-years) were higher in the gout group compared to the non-gout group (Table 4). Kaplan–Meier analysis supported this finding, indicating a higher likelihood of migraines without aura in patients with gout (*p* < 0.0001; Figure 2C).

Furthermore, crude and adjusted HRs demonstrated a significant association, with patients with gout demonstrating an elevated likelihood of developing migraines without aura ([HR, 1.26; 95% CI = 1.17–1.36; *p* < 0.001] and [aHR, 1.27; 95% CI = 1.19–1.35; *p* < 0.001], respectively). This association was consistently observed across various subgroups, including sex, income, residence, smoking, alcohol consumption, blood pressure, fasting blood glucose, total cholesterol, and CCI scores. Furthermore, patients with gout aged <70 years and those with normal weight, overweight, or obesity exhibited a higher likelihood of developing migraines without aura than the control group.

## 4. Discussion

To our knowledge, this study is the first to elucidate the relationship between gout and the onset of migraines; it analyzed distinct migraine characteristics in patients with gout. Notably, there was a higher likelihood of occurrence of migraines among patients with gout compared to those without gout. Our findings suggest that gout is an independent risk factor for the subsequent migraine occurrence; patients with gout demonstrate a 26% higher likelihood (95% CI = 1.18–1.33) of experiencing migraines, particularly the subtype without aura. Notably, the increased likelihood was consistent across specific factors, including sex, place of residence, smoking and alcohol habits, and the presence of comorbidities (hypertension, hyperglycemia, and hyperlipidemia) among individuals aged <70 years, and across different weight categories, including those with normal weight, overweight, and obesity. Our findings suggest that predicting the occurrence of migraines in patients with gout may be challenging; however, for a subset of patients with gout, lifestyle modifications, such as maintaining a lower body weight, particularly for individuals aged <70 years, could be a potential strategy for preventing migraines.

Current research on the connection between gout and migraines is limited, with only a few recently published studies suggesting a possible association. In a recent cross-sectional study involving 208 migraineurs in the United States, a correlation was found between higher migraine occurrence and increased serum urate levels of >7.8 mg/dL [24]; this suggests that elevated serum urate is a risk factor for migraines [24]. The significant association between elevated blood urate levels and an increased risk of gout [3] suggests a potential connection between the development of migraines and gout. Furthermore, a recent cross-sectional study involving 796 patients with arthritis in the United States revealed that those with arthritis, including gout, had a 1.83-fold higher risk of developing migraines (95% CI = 1.20–2.81) [20]; this finding indirectly supports the potential link between gout and migraines, consistent with our study’s observations. Additionally, the cross-sectional study revealed that the impact of arthritis, including gout, on migraines was more common among women and individuals aged ≤45 years or >65 years [20]. However, our study demonstrated that both men and women and individuals aged <70 years in the gout group exhibited a higher susceptibility to migraines than those in the matched control group. This observed elevated likelihood was consistent across groups even after analyzing the association of all potential confounding factors. This study extensively elucidates the association of gout with migraines via a research design that involved matching nationwide population-based control groups through propensity scores and fine-tuning the analysis using the overlap weighting technique to ensure an accurate balance of baseline characteristics; this approach allowed us to evaluate a wide array of potential confounding factors, including demographics, lifestyle, and comorbid conditions. Ultimately, our analysis revealed a significant association between gout and an increased likelihood of migraines.

Notably, the association between gout and migraines was most significantly linked to the occurrence of migraines without aura, which represents the majority (approximately 70%) of migraine cases [13]. Based on our study, the rate of incidence for migraines without aura was higher than that of migraines with aura among individuals with gout (5.53 vs. 0.30 per 1000 person-years). Furthermore, patients with gout had a 27% higher likelihood (95% CI = 1.19–1.35) of experiencing migraines without aura; however, this was not associated with migraines with aura. Subgroup analyses focusing on migraines without aura yielded results consistent with the findings of overall migraine, suggesting that the majority of gout-related migraines can be grouped as migraines without aura. In contrast, other inflammatory arthritic conditions, such as rheumatoid arthritis, exhibit a higher tendency for the occurrence of migraines with aura as a comorbidity [19]. The occurrence of migraines with or without aura may vary depending on the type of arthritis. Notably, a diagnosis of migraines with aura is limited due to its association with an increased risk of various comorbidities, including ischemic stroke, Parkinson’s disease, bipolar disorder, panic disorder, restless legs syndrome, and patent foramen ovale [16,35,36]. The influence of comorbidity in the occurrence of migraines without aura is significantly lower than in migraines with aura [35]. Therefore, the predominance of migraines without aura in patients with gout may be of clinical significance, warranting attention and education regarding incident migraines and their subtypes as potential gout-related comorbidities. Our results suggest that patients with gout exhibit a higher likelihood of experiencing migraines without aura; this underscores the importance of evaluating migraine subtypes in gout-related health management and education.

Understanding the potential connection between gout and migraines is a complex challenge, as it involves multifaceted factors encompassing genetics and environmental interactions. Gout and migraines share several common risk factors, including advanced age, menopause, alcohol consumption, obesity, sedentary lifestyles, hypertension, dyslipidemia, diabetes, stress, anxiety, depression, and emotional challenges [1,14,37,38]. These shared factors can lead to systemic metabolic changes, increased oxidative stress, and persistent inflammation [21,39], thereby increasing susceptibility to both conditions. For example, individuals with gout who experience gout flares, which can have adverse effects on their physical and psychosocial well-being and potentially induce stress [40], may be at risk of developing migraine headaches [41]. Stress and metabolic changes, which significantly contribute to inflammation, are known triggers for migraines and gout flares [21,39]. These conditions involve inflammatory processes mediated by similar pro-inflammatory molecules and immune system alterations, forming the basis of their association [21,42,43]. In patients with gout, the deposition and phagocytosis of monosodium urate crystals in tissues activate the NLRP3 inflammasome [44], leading to the production of critical cytokines such as IL-1β, IL-6, and tumor necrosis factor (TNF)-α [42,43], which are implicated in the occurrence of migraines [21].

Additionally, the NLRP3 inflammasome is upregulated in trigeminal ganglion neurons relevant to migraine pain [45]. Notably, elevated uric acid levels in gout trigger inflammatory responses and directly affect the central nervous system by activating inflammasomes [23,46], contributing to migraine pain development [21,45]. Increased uric acid levels from purine metabolism in gout promote the release of nitric oxide, a critical factor in migraine pathophysiology, due to the upregulation of the nitric oxide signaling cascade in patients experiencing migraines [47]. The impact of purines on various brain cell types and purinergic receptors contributes to the initiation and amplification of migraine pain [48].

While there is no specific genetic variant directly linking gout and migraines, certain genetic variants may indirectly contribute to both conditions. Notably, gout susceptibility loci, including genes such as ABCG2 and SLC2A9 (involved in urate transport), rs1260326 of GCKR (associated with glucose and lipid metabolism), rs2188380 of MYL2-CUX2 (linked to cholesterol and diabetes mellitus), and rs4073582 of CNIH-2 (regulating glutamate signaling), have been identified at the genome-wide significance level [49]. These genes could indirectly impact migraine susceptibility through metabolic and neurological pathways [49]. Furthermore, variations in CNIH-2, which modulates glutamate receptor function in neurons and glial cells [50], might indirectly influence migraine susceptibility as glutamate is significantly involved in migraine pathophysiology [51]. Additionally, MAP3K11, a member of the mitogen-activated protein kinase family, could be involved in gout pathogenesis and is vital in activating c-Jun N-terminal kinase, a stress-activated protein kinase [49]. This pathway is crucial in the molecular mechanisms underlying processes initiated by inflammation and oxidative stress [52].

The sample size, comprising 23,137 patients and 92,548 controls, drawn from a well-structured and representative nationwide healthcare database, contributes to the credibility of this study. Furthermore, the analysis was extensively adjusted to account for socioeconomic status, potential lifestyle-related risk factors, and comorbidities. Second, the utilization of data collected from all medical and clinic services in Korea allowed for the assembly of comprehensive medical records over the study period, consequently bolstering the applicability and credibility of the research findings. This comprehensive dataset may serve as a valuable resource for conducting research and analyzing health-related trends and outcomes in the Korean population. Third, a meticulously balanced selection of study and control participants achieved through propensity score matching likely fortified our research, mitigating potential selection bias and approximating the characteristics of randomized trials [53]. Despite gout being more prevalent among men and the elderly [1], we employed an overlapweighted propensity matching process. This process enabled us to perfectly and uniformly match a large number of individuals with gout (*n* = 23,137) to corresponding control participants (*n* = 92,548) in terms of age, sex, income, and residential area. As a result, our study achieved a well-balanced distribution of age, sex, and socioeconomic factors, thereby preventing potential distortions in the demographic characteristics of the research groups [54]. Additionally, the outcome based on the 16-year follow-up data may enhance reliability.

Our findings should be interpreted within the context of several limitations. First, the observational and retrospective design of this study does not permit the establishment of a direct causal relationship between gout and the onset of migraines. Additionally, we did not investigate the underlying mechanisms that could elucidate the connection between these two conditions. Second, it is important to acknowledge that our study exclusively focused on individuals aged >40 years in Korea, and we relied on diagnosis codes from Korean health insurance data. This approach may have limitations in accurately discriminating between different headache types, including the potential misclassification of tension-type headache and migraines. Consequently, certain potential confounding factors may not have been fully accounted for, and the generalizability of our findings to other demographic groups could be limited, affecting the precision of identifying patients with gout or migraines. Third, the absence of data on family history, personal genetics, or dietary factors related to gout or migraines within the KNHIS-HSC database was not considered in this study. We categorized alcohol consumption solely based on frequency (more than once a week or less) due to limitations in the available data. As a result, it is crucial to acknowledge certain limitations in our findings. These limitations include the absence of specific variables, potential geographic specificity, recall bias, and the retrospective design of the study.

## 5. Conclusions

Our study may suggest that Koreans with gout have a slightly increased likelihood of developing migraines, especially the subtype without aura, based on 16-year long-term follow-up data. This increased likelihood was consistent across various demographics, lifestyles, and socioeconomic factors; additionally, factors including the presence of comorbidities, age (<70 years) and different weight categories (including those with normal weight, overweight, and obesity) showed consistent increased likelihood. These findings imply that predicting migraine occurrence among patients with gout may pose challenges. However, for a specific subset of gout patients, there could be potential benefits in adopting lifestyle changes, such as maintaining a lower body weight, particularly for patients aged <70 years, as a potential strategy for migraine prevention. These results are vital in the management and education of patients with gout and decision-making by healthcare practitioners. Furthermore, there is a need to conduct studies to evaluate the mechanisms underlying the increased likelihood of occurrence of migraines.

## Figures and Tables

**Table 1:** Participant demographics.

Characteristics		Before Overlap Weighting Adjustment			After Overlap Weighting Adjustment		
		Gout	Control	Standardized Difference	Gout	Control	Standardized Difference
Age (n, %)				0.00			0.00
	40–44	583 (2.52)	2332 (2.52)		456 (2.51)	456 (2.51)	
	45–49	2023 (8.74)	8092 (8.74)		1578 (8.69)	1578 (8.69)	
	50–54	3490 (15.08)	13,960 (15.08)		2737 (15.07)	2737 (15.07)	
	55–59	4538 (19.61)	18,152 (19.61)		3561 (19.61)	3561 (19.61)	
	60–64	3958 (17.11)	15,832 (17.11)		3108 (17.12)	3108 (17.12)	
	65–69	3293 (14.23)	13,172 (14.23)		2591 (14.27)	2591 (14.27)	
	70–74	2556 (11.05)	10,224 (11.05)		2006 (11.05)	2006 (11.05)	
	75–79	1655 (7.15)	6620 (7.15)		1304 (7.18)	1304 (7.18)	
	80–84	795 (3.44)	3180 (3.44)		624 (3.44)	624 (3.44)	
	85+	246 (1.06)	984 (1.06)		192 (1.06)	192 (1.06)	
Sex (n, %)				0.00			0.00
	Male	18,624 (80.49)	74,496 (80.49)		14,587 (80.34)	14,587 (80.34)	
	Female	4513 (19.51)	18,052 (19.51)		3570 (19.66)	3570 (19.66)	
Income (n, %)				0.00			0.00
	1 (lowest)	3323 (14.36)	13,292 (14.36)		2613 (14.39)	2613 (14.39)	
	2	2860 (12.36)	11,440 (12.36)		2247 (12.38)	2247 (12.38)	
	3	3470 (15.00)	13,880 (15.00)		2721 (14.99)	2721 (14.99)	
	4	4870 (21.05)	19,480 (21.05)		3817 (21.02)	3817 (21.02)	
	5 (highest)	8614 (37.23)	34,456 (37.23)		6759 (37.22)	6759 (37.22)	
Region of residence (n, %)				0.00			0.00
	Urban	9861 (42.62)	39,444 (42.62)		7742 (42.64)	7742 (42.64)	
	Rural	13,276 (57.38)	53,104 (57.38)		10,416 (57.36)	10,416 (57.36)	
Obesity (n, %)				0.28			0.00
	Underweight	301 (1.30)	2341 (2.53)		265 (1.46)	265 (1.46)	
	Normal	5785 (25.00)	32,144 (34.73)		4872 (26.83)	4872 (26.83)	
	Overweight	6348 (27.44)	25,922 (28.01)		5066 (27.90)	5066 (27.90)	
	Obese I	9680 (41.84)	29,864 (32.27)		7254 (39.95)	7254 (39.95)	
	Obese II	1023 (4.42)	2277 (2.46)		701 (3.86)	701 (3.86)	
Smoking status (n, %)				0.04			0.00
	Non-smoker	12,467 (53.88)	49,897 (53.91)		9799 (53.96)	9799 (53.96)	
	Past smoker	3261 (14.09)	11,854 (12.81)		2507 (13.81)	2507 (13.81)	
	Current smoker	7409 (32.02)	30,797 (33.28)		5852 (32.23)	5852 (32.23)	
Alcohol consumption (n, %)				0.11			0.00
	<1 time a week	13,259 (57.31)	57,894 (62.56)		10,609 (58.43)	10,609 (58.43)	
	≥1 time a week	9878 (42.69)	34,654 (37.44)		7548 (41.57)	7548 (41.57)	
SBP (Mean, SD)		129.71 (16.84)	127.40 (16.23)	0.14	129.21 (14.80)	129.21 (7.34)	0.00
DBP (Mean, SD)		80.23 (11.05)	78.82 (10.50)	0.13	79.92 (9.72)	79.92 (4.73)	0.00
Fasting blood glucose (Mean, SD)		102.59 (28.11)	103.07 (30.81)	0.02	102.68 (25.39)	102.68 (12.51)	0.00
Total cholesterol (Mean, SD)		198.20 (40.42)	195.41 (38.08)	0.07	197.58 (35.59)	197.58 (17.17)	0.00
CCI score (Mean, SD)		1.20 (1.83)	0.99 (1.72)	0.12	1.15 (1.58)	1.15 (0.84)	0.00
Migraines with aura (n, %)		54 (0.23)	202 (0.22)	0.00	43 (0.24)	40 (0.22)	0.00
Migraines without aura (n, %)		946 (4.09)	3012 (3.25)	0.04	741 (4.08)	593 (3.26)	0.04

Abbreviations: SBP, systolic blood pressure; DBP, diastolic blood pressure; CCI, Charlson Comorbidity Index; SD, Standard deviation.

**Table 2:** Crude and overlap propensity score weighted hazard ratios (95% confidence interval) of gout for overall migraines, along with their subgroup analyses.

		N of Event/N of Total (%)	Follow-Up Duration (PY)	IR Per 1000 (PY)	IRD (95% CI)	Hazard Ratios for Migraine			
						Crude	*p*	Overlap Weighted Model †	*p*
Total participants									
	Gout	1000/23,137 (4.32)	170,738	5.86	1.19 (0.81–1.56)	1.25 (1.16–1.34)	<0.001 *	1.26 (1.18–1.33)	<0.001 *
	Control	3214/92,548 (3.47)	687,917	4.67		1		1	
Age < 70 years old									
	Gout	824/17,885 (4.61)	145,828	5.65	1.32 (0.93–1.71)	1.30 (1.20–1.41)	<0.001 *	1.30 (1.22–1.39)	<0.001 *
	Control	2546/71,540 (3.56)	588,045	4.33		1		1	
Age ≥ 70 years old									
	Gout	176/5252 (3.35)	24,910	7.07	0.38 (−0.76–1.52)	1.05 (0.89–1.24)	0.554	1.07 (0.94–1.23)	0.301
	Control	668/21,008 (3.18)	99,872	6.69		1		1	
Male									
	Gout	650/18,624 (3.49)	140,381	4.63	0.80 (0.43–1.17)	1.21 (1.10–1.32)	<0.001 *	1.22 (1.14–1.31)	<0.001 *
	Control	2155/74,496 (2.89)	562,459	3.83		1		1	
Female									
	Gout	350/4513 (7.76)	30,357	11.50	3.06 (1.90–4.28)	1.35 (1.20–1.53)	<0.001 *	1.32 (1.19–1.46)	<0.001 *
	Control	1059/18,052 (5.87)	125,458	8.44		1		1	
Low-income group									
	Gout	482/9653 (4.99)	67,928	7.10	1.68 (1.04–2.31)	1.30 (1.18–1.45)	<0.001 *	1.31 (1.20–1.43)	<0.001 *
	Control	1487/38,612 (3.85)	274,345	5.42		1		1	
High-income group									
	Gout	518/13,484 (3.84)	102,810	5.04	0.86 (0.41–1.31)	1.20 (1.09–1.33)	<0.001 *	1.21 (1.11–1.31)	<0.001 *
	Control	1727/53,936 (3.20)	413,572	4.18		1		1	
Urban resident									
	Gout	383/9861 (3.88)	74,339	5.15	0.85 (0.32–1.39)	1.19 (1.07–1.34)	0.002 *	1.20 (1.09–1.32)	<0.001 *
	Control	1289/39,444 (3.27)	299,849	4.30		1		1	
Rural resident									
	Gout	617/13,276 (4.65)	96,399	6.40	1.44 (0.93–1.95)	1.29 (1.17–1.41)	<0.001 *	1.29 (1.20–1.39)	<0.001 *
	Control	1925/53,104 (3.62)	388,068	4.96		1		1	
Underweight									
	Gout	11/301 (3.65)	1845	5.96	1.16 (−2.23–4.56)	1.22 (0.65–2.31)	0.533	1.20 (0.79–1.84)	0.398
	Control	72/2341 (3.08)	14,997	4.80		1		1	
Normal weight									
	Gout	263/5785 (4.55)	41,140	6.39	1.54 (0.80–2.29)	1.30 (1.14–1.49)	<0.001 *	1.24 (1.13–1.38)	<0.001 *
	Control	1148/32,144 (3.57)	236,931	4.85		1		1	
Overweight									
	Gout	269/6348 (4.24)	47,112	5.71	1.04 (0.34–1.74)	1.22 (1.06–1.39)	0.005 *	1.22 (1.10–1.37)	<0.001 *
	Control	915/25,922 (3.53)	196,033	4.67		1		1	
Obese									
	Gout	457/10,703 (4.27)	80,641	5.67	1.17 (0.62–1.72)	1.26 (1.13–1.41)	<0.001 *	1.30 (1.18–1.43)	<0.001 *
	Control	1079/32,141 (3.36)	239,956	4.50		1		1	
Non-smoker									
	Gout	670/12,467 (5.37)	92,322	7.26	1.63 (1.07–2.18)	1.29 (1.18–1.41)	<0.001 *	1.29 (1.20–1.38)	<0.001 *
	Control	2070/49,897 (4.15)	367,435	5.63		1		1	
Past and current smoker									
	Gout	330/10,670 (3.09)	78,416	4.21	0.64 (0.16–1.11)	1.17 (1.04–1.32)	0.011 *	1.19 (1.08–1.32)	<0.001 *
	Control	1144/42,651 (2.68)	320,482	3.57		1		1	
Alcohol consumption < 1 time a week									
	Gout	682/13,259 (5.14)	95,776	7.12	1.72 (1.19–2.25)	1.31 (1.20–1.43)	<0.001 *	1.26 (1.18–1.35)	<0.001 *
	Control	2304/57,894 (3.98)	426,697	5.40		1		1	
Alcohol consumption ≥ 1 time a week									
	Gout	318/9878 (3.22)	74,962	4.24	0.76 (0.27–1.25)	1.22 (1.07–1.38)	0.002 *	1.23 (1.11–1.38)	<0.001 *
	Control	910/34,654 (2.63)	261,220	3.48		1		1	
SBP < 140 mmHg and DBP < 90 mmHg									
	Gout	670/15,717 (4.26)	110,255	6.08	1.34 (0.88–1.80)	1.27 (1.17–1.39)	<0.001 *	1.24 (1.16–1.33)	<0.001 *
	Control	2330/68,278 (3.41)	492,071	4.74		1		1	
SBP ≥ 140 mmHg or DBP ≥ 90 mmHg									
	Gout	330/7420 (4.45)	60,483	5.46	0.95 (0.31–1.57)	1.21 (1.07–1.37)	0.003 *	1.29 (1.15–1.44)	<0.001 *
	Control	884/24,270 (3.64)	195,846	4.51		1		1	
Fasting blood glucose < 100 mg/dL									
	Gout	650/13,015 (4.99)	101,768	6.39	1.45 (0.96–1.94)	1.29 (1.18–1.41)	<0.001 *	1.30 (1.21–1.39)	<0.001 *
	Control	2096/53,644 (3.91)	424,563	4.94		1		1	
Fasting blood glucose ≥ 100 mg/dL									
	Gout	350/10,122 (3.46)	68,970	5.07	0.82 (0.27–1.39)	1.19 (1.06–1.35)	0.004 *	1.19 (1.07–1.31)	<0.001 *
	Control	1118/38,904 (2.87)	263,354	4.25		1		1	
Total cholesterol < 200 mg/dL									
	Gout	521/12,436 (4.19)	86,682	6.01	1.26 (0.73–1.78)	1.25 (1.14–1.38)	<0.001 *	1.24 (1.14–1.34)	<0.001 *
	Control	1791/52,406 (3.42)	376,658	4.75		1		1	
Total cholesterol ≥ 200 mg/dL									
	Gout	479/10,701 (4.48)	84,056	5.70	1.13 (0.60–1.66)	1.25 (1.12–1.38)	<0.001 *	1.28 (1.17–1.40)	<0.001 *
	Control	1423/40,142 (3.54)	311,259	4.57		1		1	
CCI scores = 0									
	Gout	466/12,458 (3.74)	92,953	5.01	1.03 (0.57–1.49)	1.25 (1.13–1.39)	<0.001 *	1.29 (1.19–1.41)	<0.001 *
	Control	1704/56,064 (3.04)	427,756	3.98		1		1	
CCI scores = 1									
	Gout	221/3994 (5.53)	29,611	7.46	1.56 (0.55–2.58)	1.26 (1.08–1.47)	0.003 *	1.28 (1.12–1.46)	<0.001 *
	Control	635/14,591 (4.35)	107,620	5.90		1		1	
CCI scores ≥ 2									
	Gout	313/6685 (4.68)	48,174	6.50	0.76 (−0.03–1.55)	1.14 (1.00–1.29)	0.050	1.13 (1.01–1.26)	0.038 *
	Control	875/21,893 (4.00)	152,541	5.74		1		1	

Abbreviation: IR, incidence rate; CI, confidence interval; IRD, incidence rate difference; PY, person-year; SBP, systolic blood pressure; DBP, diastolic blood pressure; CCI, Charlson Comorbidity Index. * Significance at *p* < 0.05. † Adjusted for age, sex, income, region of residence, obesity, smoking, alcohol consumption, systolic blood pressure, diastolic blood pressure, fasting blood glucose, total cholesterol, and CCI scores.

**Table 3:** Crude and overlap propensity score weighted hazard ratios (95% confidence interval) of gout for the subtype of migraines with aura, along with their subgroup analyses.

		N of Event/N of Total (%)	Follow-Up Duration (PY)	IR Per 1000 (PY)	IRD (95% CI)	Hazard Ratios for Migraines with Aura			
						Crude	*p*	Overlap Weighted Model †	*p*
Total participants									
	Gout	54/23,137 (0.23)	177,158	0.30	0.01 (−0.07–0.11)	1.07 (0.79–1.44)	0.673	1.08 (0.84–1.37)	0.554
	Control	202/92,548 (0.22)	707,898	0.29		1		1	
Age < 70 years old									
	Gout	50/17,885 (0.28)	151,340	0.33	0.05 (−0.04–0.15)	1.19 (0.87–1.63)	0.284	1.17 (0.91–1.52)	0.229
	Control	168/71,540 (0.23)	604,676	0.28		1		1	
Age ≥ 70 years old									
	Gout	4/5252 (0.08)	25,818	0.15	−0.18 (−0.41–0.06)	0.47 (0.17–1.32)	0.153	0.52 (0.25–1.11)	0.092
	Control	34/21,008 (0.16)	103,222	0.33		1		1	
Male									
	Gout	31/18,624 (0.17)	144,493	0.21	−0.02 (−0.10–0.07)	0.94 (0.64–1.39)	0.764	1.01 (0.74–1.38)	0.949
	Control	131/74,496 (0.18)	575,410	0.23		1		1	
Female									
	Gout	23/4513 (0.51)	32,665	0.70	0.16 (−0.12–0.46)	1.31 (0.82–2.09)	0.263	1.27 (0.86–1.89)	0.230
	Control	71/18,052 (0.39)	132,488	0.54		1		1	
Low-income group									
	Gout	28/9653 (0.29)	70,996	0.39	0.03 (−0.12–0.19)	1.09 (0.72–1.66)	0.675	1.12 (0.80–1.58)	0.493
	Control	102/38,612 (0.26)	283,343	0.36		1		1	
High-income group									
	Gout	26/13,484 (0.19)	106,162	0.24	0.00 (−0.09–0.11)	1.04 (0.68–1.60)	0.861	1.02 (0.72–1.44)	0.924
	Control	100/53,936 (0.19)	424,555	0.24		1		1	
Urban resident									
	Gout	12/9861 (0.12)	76,933	0.16	−0.10 (−0.23–0.02)	0.60 (0.33–1.10)	0.098	0.61 (0.39–0.97)	0.035 *
	Control	80/39,444 (0.20)	307,749	0.26		1		1	
Rural resident									
	Gout	42/13,276 (0.32)	100,225	0.42	0.12 (−0.01–0.24)	1.37 (0.97–1.95)	0.076	1.36 (1.02–1.83)	0.037 *
	Control	122/53,104 (0.23)	400,149	0.30		1		1	
Underweight									
	Gout	1/301 (0.33)	1892	0.53	0.40 (−0.23–1.03)	4.10 (0.37–45.3)	0.25	20.3 (1.52–270)	0.023 *
	Control	2/2341 (0.09)	15,421	0.13		1		1	
Normal weight									
	Gout	19/5785 (0.33)	42,844	0.44	0.11 (−0.08–0.31)	1.34 (0.81–2.21)	0.251	1.32 (0.90–1.93)	0.154
	Control	80/32,144 (0.25)	244,054	0.33		1		1	
Overweight									
	Gout	14/6348 (0.22)	48,864	0.29	0.07 (−0.09–0.22)	1.28 (0.70–2.33)	0.418	1.34 (0.81–2.20)	0.254
	Control	45/25,922 (0.17)	201,869	0.22		1		1	
Obese									
	Gout	20/10,703 (0.19)	83,558	0.24	−0.06 (−0.20–0.07)	0.79 (0.48–1.29)	0.346	0.80 (0.53–1.21)	0.284
	Control	75/32,141 (0.23)	246,554	0.30		1		1	
Non-smoker									
	Gout	35/12,467 (0.28)	96,664	0.36	0.00 (−0.13–0.14)	1.01 (0.70–1.46)	0.967	1.00 (0.74–1.34)	0.979
	Control	137/49,897 (0.27)	380,696	0.36		1		1	
Past and current smoker									
	Gout	19/10,670 (0.18)	80,494	0.24	0.04 (−0.07–0.15)	1.18 (0.71–1.97)	0.523	1.31 (0.86–2.01)	0.207
	Control	65/42,651 (0.15)	327,202	0.20		1		1	
Alcohol consumption < 1 time a week									
	Gout	37/13,259 (0.28)	100,183	0.37	0.02 (−0.11–0.15)	1.05 (0.74–1.51)	0.774	1.02 (0.77–1.35)	0.879
	Control	154/57,894 (0.27)	441,348	0.35		1		1	
Alcohol consumption ≥ 1 time a week									
	Gout	17/9878 (0.17)	76,975	0.22	0.04 (−0.07–0.15)	1.23 (0.71–2.14)	0.465	1.25 (0.78–2.00)	0.357
	Control	48/34,654 (0.14)	266,550	0.18		1		1	
SBP < 140 mmHg and DBP < 90 mmHg									
	Gout	38/15,717 (0.24)	114,373	0.33	0.02 (−0.09–0.14)	1.07 (0.75–1.53)	0.702	1.04 (0.79–1.38)	0.782
	Control	156/68,278 (0.23)	506,170	0.31		1		1	
SBP ≥ 140 mmHg or DBP ≥ 90 mmHg									
	Gout	16/7420 (0.22)	62,785	0.25	0.02 (−0.11–0.16)	1.12 (0.63–1.98)	0.699	1.15 (0.71–1.86)	0.572
	Control	46/24,270 (0.19)	201,728	0.23		1		1	
Fasting blood glucose < 100 mg/dL									
	Gout	34/13,015 (0.26)	106,061	0.32	−0.01 (−0.13–0.11)	0.97 (0.67–1.41)	0.861	0.96 (0.72–1.29)	0.789
	Control	145/53,644 (0.27)	437,864	0.33		1		1	
Fasting blood glucose ≥ 100 mg/dL									
	Gout	20/10,122 (0.20)	71,097	0.28	0.07 (−0.05–0.19)	1.33 (0.80–2.22)	0.270	1.35 (0.88–2.08)	0.169
	Control	57/38,904 (0.15)	270,034	0.21		1		1	
Total cholesterol < 200 mg/dL									
	Gout	28/12,436 (0.23)	90,015	0.31	−0.01 (−0.14–0.12)	0.96 (0.64–1.45)	0.843	0.94 (0.68–1.30)	0.708
	Control	125/52,406 (0.24)	387,528	0.32					
Total cholesterol ≥ 200 mg/dL									
	Gout	26/10,701 (0.24)	87,143	0.30	0.06 (−0.06–0.18)	1.24 (0.80–1.94)	0.335	1.30 (0.90–1.89)	0.165
	Control	77/40,142 (0.19)	320,370	0.24		1		1	
CCI scores = 0									
	Gout	19/12,458 (0.15)	95,999	0.20	−0.05 (−0.16–0.06)	0.79 (0.48–1.28)	0.331	0.83 (0.58–1.20)	0.321
	Control	110/56,064 (0.20)	438,344	0.25		1		1	
CCI scores = 1									
	Gout	19/3994 (0.48)	31,031	0.61	0.26 (0.01–0.52)	1.76 (1.01–3.04)	0.044 *	1.65 (1.01–2.68)	0.045 *
	Control	39/14,591 (0.27)	111,582	0.35		1		1	
CCI scores ≥ 2									
	Gout	16/6685 (0.24)	50,128	0.32	−0.02 (−0.20–0.17)	0.96 (0.55–1.67)	0.873	0.93 (0.58–1.50)	0.769
	Control	53/21,893 (0.24)	157,972	0.34		1		1	

Abbreviation: IR, incidence rate; CI, confidence interval; IRD, incidence rate difference; PY, person-year; SBP, systolic blood pressure; DBP, diastolic blood pressure; CCI, Charlson Comorbidity Index. * Significance at *p* < 0.05. † Adjusted for age, sex, income, region of residence, obesity, smoking, alcohol consumption, systolic blood pressure, diastolic blood pressure, fasting blood glucose, total cholesterol, and CCI scores.

**Table 4:** Crude and overlap propensity score weighted hazard ratios (95% confidence interval) of gout for the subtype of migraines without aura, along with their subgroup analyses.

		N of Event/N of Total (%)	Follow-Up Duration (PY)	IR Per 1000 (PY)	IRD (95% CI)	Hazard Ratios for Migraines without Aura			
						Crude	*p*	Overlap Weighted Model †	*p*
Total participants									
	Gout	946/23,137 (4.09)	171,117	5.53	1.26 (1.17–1.36)	1.26 (1.17–1.36)	<0.001 *	1.27 (1.19–1.35)	<0.001 *
	Control	3012/92,548 (3.25)	689,450	4.37					
Age < 70 years old									
	Gout	774/17,885 (4.33)	146,171	5.30	1.27 (0.89–1.64)	1.31 (1.21–1.42)	<0.001 *	1.31 (1.22–1.40)	<0.001 *
	Control	2378/71,540 (3.32)	589,356	4.03					
Age ≥ 70 years old									
	Gout	172/5252 (3.27)	24,946	6.89	0.56 (−0.55–1.67)	1.08 (0.91–1.28)	0.358	1.10 (0.96–1.26)	0.173
	Control	634/21,008 (3.02)	100,094	6.33					
Male									
	Gout	619/18,624 (3.32)	140,582	4.40	0.81 (0.45–1.17)	1.22 (1.12–1.34)	<0.001 *	1.24 (1.15–1.33)	<0.001 *
	Control	2024/74,496 (2.72)	563,424	3.59					
Female									
	Gout	327/4513 (7.25)	30,535	10.70	2.86 (1.72–4.02)	1.36 (1.20–1.54)	<0.001 *	1.32 (1.19–1.46)	<0.001 *
	Control	988/18,052 (5.47)	126,026	7.84					
Low-income group									
	Gout	454/9653 (4.70)	68,129	6.66	1.62 (1.01–2.24)	1.32 (1.19–1.47)	<0.001 *	1.32 (1.21–1.45)	<0.001 *
	Control	1385/38,612 (3.59)	275,061	5.04					
High-income group									
	Gout	492/13,484 (3.65)	102,988	4.78	0.85 (0.41–1.29)	1.21 (1.10–1.34)	<0.001 *	1.22 (1.12–1.32)	<0.001 *
	Control	1627/53,936 (3.02)	414,389	3.93					
Urban resident									
	Gout	371/9861 (3.76)	74,412	4.99	0.97 (0.44–1.48)	1.24 (1.10–1.39)	<0.001 *	1.24 (1.12–1.36)	<0.001 *
	Control	1209/39,444 (3.07)	300,417	4.02					
Rural resident									
	Gout	575/13,276 (4.33)	96,705	5.95	1.32 (0.82–1.80)	1.28 (1.16–1.41)	<0.001 *	1.29 (1.19–1.39)	<0.001 *
	Control	1803/53,104 (3.40)	389,033	4.63					
Underweight									
	Gout	10/301 (3.32)	1854	5.39	0.73 (−2.59–4.05)	1.14 (0.59–2.22)	0.695	1.12 (0.72–1.73)	0.627
	Control	70/2341 (2.99)	15,015	4.66					
Normal weight									
	Gout	244/5785 (4.22)	41,289	5.91	1.41 (0.70–2.13)	1.30 (1.13–1.49)	<0.001 *	1.24 (1.11–1.37)	<0.001 *
	Control	1068/32,144 (3.32)	237,496	4.50					
Overweight									
	Gout	255/6348 (4.02)	47,185	5.40	0.97 (0.29–1.66)	1.21 (1.06–1.40)	0.006 *	1.22 (1.09–1.36)	<0.001 *
	Control	870/25,922 (3.36)	196,392	4.43					
Obese									
	Gout	437/10,703 (4.08)	80,789	5.41	1.24 (0.70–1.77)	1.30 (1.16–1.45)	<0.001 *	1.33 (1.21–1.47)	<0.001 *
	Control	1004/32,141 (3.12)	240,547	4.17					
Non-smoker									
	Gout	635/12,467 (5.09)	92,582	6.86	1.61 (1.07–2.15)	1.31 (1.20–1.43)	<0.001 *	1.31 (1.21–1.41)	<0.001 *
	Control	1933/49,897 (3.87)	368,467	5.25					
Past and current smoker									
	Gout	311/10,670 (2.91)	78,535	3.96	0.60 (0.14–1.06)	1.17 (1.03–1.33)	0.014 *	1.19 (1.07–1.32)	0.001 *
	Control	1079/42,651 (2.53)	320,983	3.36					
Alcohol consumption < 1 time a week									
	Gout	645/13,259 (4.86)	96,064	6.71	1.69 (1.18–2.20)	1.33 (1.22–1.45)	<0.001 *	1.28 (1.19–1.37)	<0.001 *
	Control	2150/57,894 (3.71)	427,881	5.02					
Alcohol consumption ≥ 1 time a week									
	Gout	301/9878 (3.05)	75,053	4.01	0.71 (0.24–1.19)	1.22 (1.07–1.39)	0.003 *	1.23 (1.10–1.38)	<0.001 *
	Control	862/34,654 (2.49)	261,569	3.30					
SBP < 140 mmHg and DBP < 90 mmHg									
	Gout	632/15,717 (4.02)	110,514	5.72	1.31 (0.87–1.76)	1.29 (1.18–1.41)	<0.001 *	1.26 (1.17–1.35)	<0.001 *
	Control	2174/68,278 (3.18)	493,240	4.41					
SBP ≥ 140 mmHg or DBP ≥ 90 mmHg									
	Gout	314/7420 (4.23)	60,603	5.18	0.91 (0.30–1.52)	1.21 (1.07–1.38)	0.003 *	1.30 (1.16–1.45)	<0.001 *
	Control	838/24,270 (3.45)	196,210	4.27					
Fasting blood glucose < 100 mg/dL									
	Gout	616/13,015 (4.73)	102,011	6.04	1.46 (0.98–1.93)	1.31 (1.20–1.44)	<0.001 *	1.32 (1.23–1.42)	<0.001 *
	Control	1951/53,644 (3.64)	425,646	4.58					
Fasting blood glucose ≥ 100 mg/dL									
	Gout	330/10,122 (3.26)	69,106	4.78	0.76 (0.21–1.29)	1.19 (1.05–1.34)	0.007 *	1.18 (1.06–1.31)	0.002 *
	Control	1061/38,904 (2.73)	263,804	4.02					
Total cholesterol < 200 mg/dL									
	Gout	493/12,436 (3.96)	86,895	5.67	1.26 (0.76–1.76)	1.28 (1.15–1.41)	<0.001 *	1.26 (1.16–1.37)	<0.001 *
	Control	1666/52,406 (3.18)	377,592	4.41					
Total cholesterol ≥ 200 mg/dL									
	Gout	453/10,701 (4.23)	84,222	5.38	1.06 (0.55–1.58)	1.25 (1.12–1.39)	<0.001 *	1.28 (1.17–1.40)	<0.001 *
	Control	1346/40,142 (3.35)	311,858	4.32					
CCI scores = 0									
	Gout	447/12,458 (3.59)	93,084	4.80	1.08 (0.64–1.53)	1.29 (1.16–1.43)	<0.001 *	1.33 (1.22–1.44)	<0.001 *
	Control	1594/56,064 (2.84)	428,607	3.72					
CCI scores = 1									
	Gout	202/3994 (5.06)	29,742	6.79	1.27 (0.29–2.24)	1.23 (1.05–1.44)	0.012 *	1.25 (1.09–1.43)	0.001 *
	Control	596/14,591 (4.08)	107,880	5.52					
CCI scores ≥ 2									
	Gout	297/6685 (4.44)	48,291	6.15	0.78 (0.01–1.54)	1.15 (1.01–1.31)	0.039 *	1.14 (1.01–1.28)	0.028 *
	Control	822/21,893 (3.75)	152,963	5.37					

Abbreviation: IR, incidence rate; CI, confidence interval; IRD, incidence rate difference; PY, person-year; SBP, systolic blood pressure; DBP, diastolic blood pressure; CCI, Charlson Comorbidity Index. * Significance at *p* < 0.05. † Adjusted for age, sex, income, region of residence, obesity, smoking, alcohol consumption, systolic blood pressure, diastolic blood pressure, fasting blood glucose, total cholesterol, and CCI scores.

## Data Availability

All data are available from the database of the National Health Insurance Sharing Service (NHISS) https://nhiss.nhis.or.kr/ (accessed on 1 March 2023). NHISS allows access to these data for any researcher who promises to follow the research ethics at some processing charge.
